# Stereoselective Synthesis of Topologically Chiral Knots and Links: Synthesis and Applications

**DOI:** 10.3390/molecules31111953

**Published:** 2026-06-04

**Authors:** Benteng Ma, Yan Sun, Haifeng Tian, Xiao Zhang, Yuheng Ju, Saiwen Gao, Lin Wu

**Affiliations:** 1 High Purity Chemistry Science and Technology Innovation Center of Jilin Province, Center of Analysis and Measurement, Jilin University of Chemical Technology, 45 Chengde Street, Jilin 132022, China; 13331629907@163.com (B.M.); 15512268629@163.com (H.T.); zx2561094342@163.com (X.Z.); juyuheng_2002@163.com (Y.J.); 18103789927@163.com (S.G.); 2 Key Laboratory of Chemical Waste Resource Utilization of Jilin Province, School of Resources and Environment Engineering, Jilin University of Chemical Technology, 45 Chengde Street, Jilin 132022, China; sunyan3@jluct.edu.cn

**Keywords:** topological chirality, molecular knots, molecular links

## Abstract

Topologically chiral molecular knots and links represent a unique class of stereochemical architectures in which handedness is encoded by the global crossing pattern of an entangled framework rather than by a local stereogenic element. Their configurational robustness and shape-persistent chiral environments make them promising platforms for molecular recognition, catalysis, chiroptical response, and spin-selective transport. This review summarizes recent progress in the stereoselective synthesis of topologically chiral knots and links, with emphasis on chirality transfer from point, axial and helical elements into persistent topological handedness. Major synthetic strategies are organized into helicity-driven approaches, template-free dynamic systems, coordination-driven self-assembly, and chiral self-sorting. The applications of knots in host–guest confinement, asymmetric catalysis, chiral recognition, and spin-selective transport are also discussed.

## 1. Introduction

Knotting represents a ubiquitous and functionally relevant structural motif across length scales, ranging from macroscopic ropes and textiles to DNA [1,2,3], proteins [4,5], and synthetic polymer chains [6,7,8]. Yet, whereas random entanglements arise readily in flexible long-chain systems, the controlled formation of discrete molecular knots with well-defined topology remains a formidable synthetic challenge [9,10,11]. Since the first synthetic trefoil knot was reported, the field has advanced from isolated demonstrations of simple topologies to increasingly sophisticated strategies capable of generating higher-order knots and links through metal templation, hydrophobic assembly, strand folding, and dynamic covalent capture. These developments have transformed molecular knotting from a conceptual curiosity into an emerging branch of molecular nanotopology.

**Figure 1:**
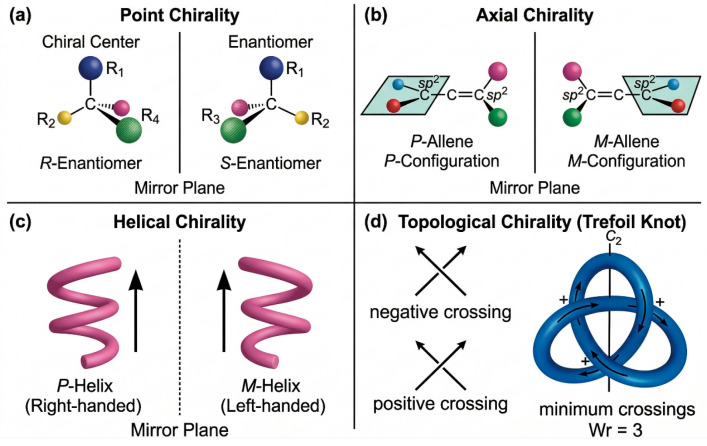
Schematic comparison of common stereogenic elements and topological chirality, (**a**) point chirality, (**b**) axial chirality, (**c**) helical chirality, (**d**) topological chirality, "+" signs mark positive topological crossings, paired with the schematic negative crossing symbol on the left panel for contrast of two fundamental knot crossing types.

More broadly, the origin, amplification, and transmission of molecular chirality have long attracted broad interest, as exemplified by studies on asymmetric autocatalysis, homochirality, and the development of molecular chirality research [12,13]. Among the stereochemical consequences of molecular entanglement, topological chirality occupies a unique position [14]. In topologically chiral molecules, handedness arises not from local stereogenic centers, chiral axes, or helical conformations, but from the global connectivity and crossing pattern of the entangled framework (Figure 1). Consequently, mirror-image topological enantiomers cannot interconvert without bond cleavage or strand crossing, conferring exceptional configurational robustness. This distinguishes topological chirality from more conventional stereochemical elements and makes it especially attractive as a persistent chiral scaffold for molecular recognition [15], catalysis [16,17,18,19], chiroptical response, and spin-selective transport [20,21].

To systematically describe and classify these topologically distinct structures, the Alexander–Briggs notation [22] is widely adopted in the field. This mathematical notation provides a standardized way to name knots and links, written in the form $X_{z}^{y}$, where Xcorresponds to the number of crossings within the system, y is the number of discrete components (loops), and z is the order of the knot used to distinguish a given topology from others with the same X and y descriptors. According to this convention, common knots discussed in this review, such as the trefoil and cinquefoil knots, are denoted $3_{1}$ and $5_{1}$, respectively.

Related macrocyclic systems, such as inherently chiral or stereoactive calixarenes, can display conformational or inherent chirality and have been widely studied in supramolecular recognition [23], sensing [24], catalysis [25], and stereoelectrochemistry [26]. To the best of our knowledge, however, calixarene chirality has not yet been directly exploited for the stereoselective construction of molecular knots. Calixarene derivatives have instead been more commonly used as macrocyclic components in simpler mechanically interlocked architectures, including rotaxanes, polyrotaxanes, and catenanes, where their cavities, conformational rigidity, and recognition ability facilitate threading and interlocking [27,28,29,30,31,32,33]. These systems provide valuable background for understanding macrocyclic chirality and mechanical bonding, but they generally lack the nontrivial over-and-under entanglement that defines molecular knots and topologically chiral links. Therefore, calixarenes are not discussed in detail in this review, which focuses on molecular knots and links/catenanes whose handedness is encoded by global crossing patterns.

In this context, the key question is no longer simply how to tie molecular knots, but how to do so in a stereocontrolled and ultimately programmable manner. This review therefore focuses on the stereoselective synthesis of topologically chiral structures, with emphasis on the major design strategies used to achieve topological stereocontrol, and the emerging functions of knots and related topological links [34,35,36].

## 2. Stereoselective Synthesis of Topologically Chiral Knots and Links

Stereoselective construction of topologically chiral architectures stands among the most demanding objectives at the interface of topology, supramolecular chemistry, and stereochemistry. Because topological enantiomers are configurationally stable without bond cleavage, stereocontrol must be enforced during folding, assembly, and covalent fixation. Successful synthesis therefore requires not merely entanglement but stereodirected assembly that selectively encodes one topological handedness.

### 2.1. Helicity-Driven Strategies

Helical intermediates provide one of the most effective platforms for translating local stereochemical information into global topological chirality [37,38,39,40,41,42]. In such systems, the helicity of a helicate precursor predetermines the crossing pattern, which can subsequently be trapped through covalent closure or preserved in the final entangled architecture. Depending on the topology of the precursor, these approaches can be broadly divided into linear helicate-based and circular helicate-based construction, both of which have played central roles in the development of molecular knots and links.

A central strategy is to preinstall covalent chirality into molecular precursors and transfer this local stereochemical information into a global topological stereogenic element during self-assembly and fixation. In such systems, covalent chirality actively biases the handedness of helical [43], entangled, or interlocked intermediates [44]. Once topology is covalently fixed, the initially local stereochemical information becomes locked into the overall architecture, enabling stepwise chiral amplification from the molecular to the topological level.

#### 2.1.1. Linear Helicate

Linear helicates provided some of the earliest examples of stereochemical control in molecular knot synthesis. In these systems, metal ions organize two ligand strands into a double helix, while chiral ligands determine the preferred helicity. Ring closure then converts the double-helical precursor into a trefoil knot. Although this strategy is most naturally suited to relatively simple topologies, it established the principle that molecular chirality can be deliberately transformed into topological chirality.

**Figure 2:**
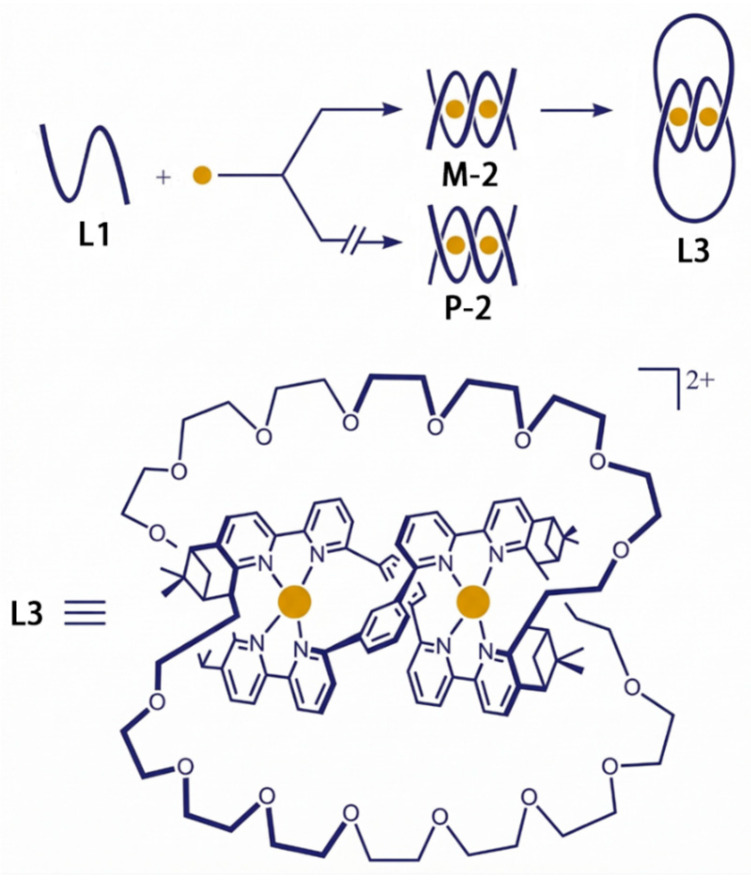
Stereoselective formation of a single double-stranded helicate during Cu(I) complexation of a chiral molecular thread. Reproduced with permission from Ref. [45], copyright 2004 Angew. Chem. Int. Ed.

Sauvage, von Zelewsky and co-workers reported a landmark example in which pinene-derived CHIRAGEN bipyridine ligands assemble with Cu(I) to form a single-handed dinuclear double helicate **M-2** (Figure 2) [45]. Diastereoselective alkylation of the ligand precursor afforded the bis-alkene ligand in 75% yield. Upon addition of Cu(I), two strands rapidly formed the double-helical complex in essentially quantitative yield, with more than 95% of the product assigned to the desired helicate by NMR spectroscopy. Ring-closing metathesis (RCM) covalently captured the preorganized topology **L3** in 74% total yield, followed by hydrogenation and demetallation to give the corresponding organic trefoil knot. The helicate and knot were characterized by UV, ESI-MS, and circular dichroism (CD), which together supported the transfer of ligand chirality into a single topological handedness.

This synthesis is significant because the topological enantiomer was not obtained by resolution of a racemate. Instead, the assembly pathway itself was stereochemically programmed. The work therefore established a prototype for helicity-driven topological stereoinduction, in which local covalent chirality is amplified through metal coordination and ultimately fixed as persistent topological chirality.

Subsequent single-strand folding strategies built on the same idea, showing that a flexible molecular strand can be preorganized into a knot-like conformation before cyclization [46,47]. These systems highlight the importance of combining ligand design, metal coordination and dynamic error correction. Nevertheless, linear helicate-derived approaches remain most powerful for trefoil-type architectures, whereas circular helicates offer a broader route to higher-order topologies.

#### 2.1.2. Circular Helicate

Circular helicates provide a more preorganized and scalable platform for constructing complex molecular knots and links. In these assemblies, multiple ligand strands wrap around a cyclic array of metal ions, and the crossing pattern is encoded before covalent capture. The helicity of the circular precursor can be predetermined by ligand chirality, metal-center configuration or the order of metal-ion addition, allowing efficient conversion of covalent or coordination chirality into topological chirality.

A representative advance was reported by Leigh and co-workers, who showed that different knot topologies can be tied from the same molecular strand by controlling the sequence of metal-ion-induced folding events [48]. The pentatopic strand contains alternating pdc and dpp coordination sites: Lu(III) organizes the pdc units into a chiral circular helicate, whereas Cu(I) generates a clasp-like crossing at the dpp sites.

**Figure 3:**
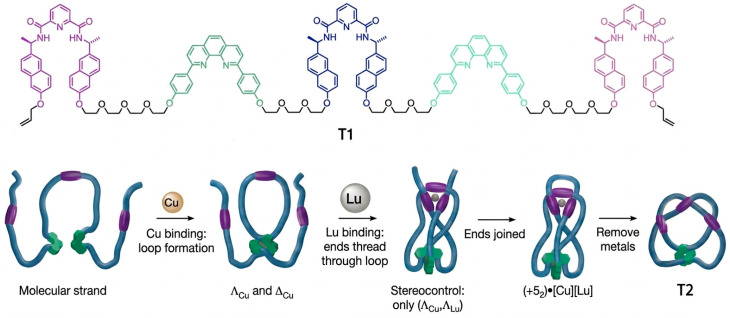
Pathway-controlled folding of a molecular strand into a three-twist (5_2_) knot through metal-ion-induced folding and entanglement. Reproduced with permission from Ref. [48], copyright 2015 J. Am. Chem. Soc.

Sequential Cu(I) then Lu(III) complexation folds the strand into a precursor of the (+5_2_) three-twist knot (Figure 3). Cu(I) complexation proceeded in 93% yield, and subsequent Lu(III)-induced threading followed by ring-closing metathesis afforded the bimetallic (+5_2_) knot in 14% yield over two steps. After covalent capture, demetallation gave the corresponding metal-free topoisomers: an unknot (0_1_) from direct macrocyclization in 35% yield, a trefoil knot (3_1_) in 57% yield and a three-twist knot **T2** (5_2_) in 66% yield. Mass spectrometry confirmed the identical molecular mass of the topoisomers, whereas DOSY and variable-temperature NMR spectroscopy distinguished their sizes and dynamics. This work marked a transition from static template control to pathway-dependent molecular knotting.

**Figure 4:**
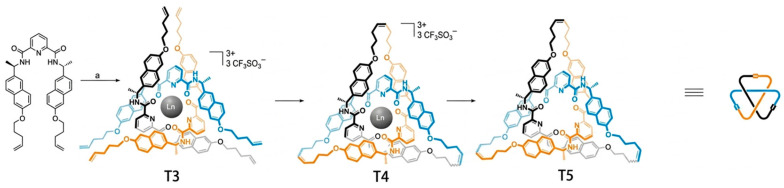
Lanthanide-templated synthesis of a single-handed trefoil knot (**T5**) via helicate formation. Reproduced with permission from Ref. [49], copyright 2015 J. Am. Chem. Soc.

Leigh and co-workers also developed an efficient lanthanide-templated route to trefoil knots of single handedness (Figure 4) [49]. Enantiopure 2,6-pyridinedicarboxamide ligands bearing point chirality assemble around Eu(III) or Lu(III) to form entangled 3:1 ligand-metal complexes of defined helicity. Treatment of the (R,R)-ligand with Ln(CF_3_SO_3_)_3_ gave the corresponding helicates **T3** in high yield, 83% for Eu(III) and 89% for Lu(III), and the single-handed nature of the assemblies was supported by symmetric NMR spectra and ESI-MS analysis.

RCM of the lanthanide helicates generated a mixture of a trefoil knot **T4** and an unknot macrocycle. Selective demetallation of the unknot with Na_5_DTPA enabled isolation of the Lu(III) trefoil **T5** in 62% yield and the Eu(III) analogue in 55% yield; further demetallation with tetraethylammonium fluoride furnished the wholly organic trefoil knot in 74% yield. The products were characterized by NMR spectroscopy, ESI-MS, single-crystal X-ray diffraction, UV–vis spectroscopy, and CD spectroscopy. X-ray structures unambiguously confirmed the opposite Lambda and Delta trefoil topologies, while CD spectroscopy showed that the topological chirality of the knot generated a stronger chiroptical response than the corresponding unknot macrocycle.

**Figure 5:**
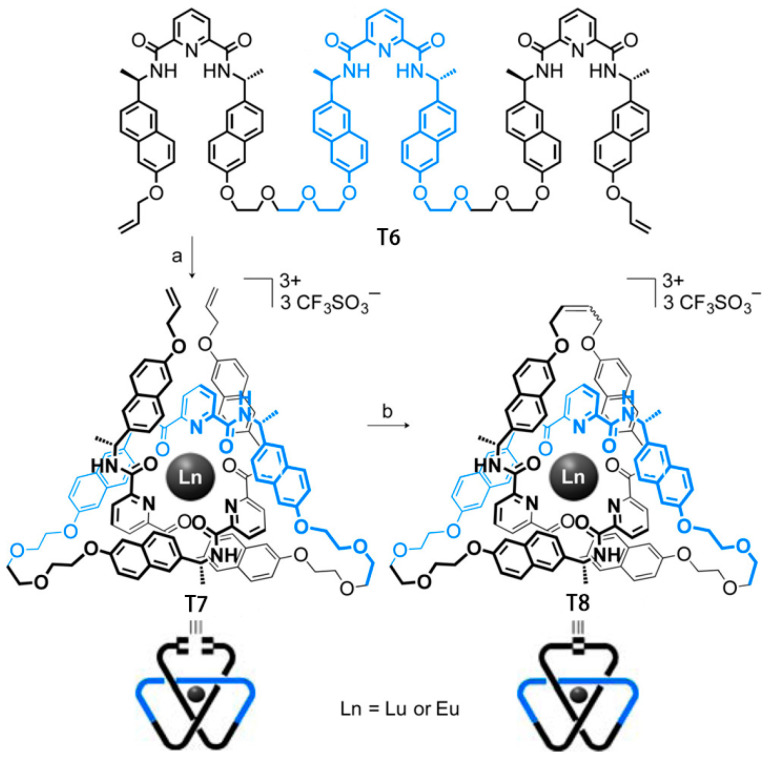
Stereoselective synthesis of an enantiopure trefoil knot via lanthanide-templated single-strand folding. Reproduced with permission from Ref. [16], copyright 2016 J. Am. Chem. Soc.

The lanthanide-templated strategy was further extended to single-strand folding by Leigh and co-workers (Figure 5) [8]. A tris(2,6-pyridinedicarboxamide) oligomer **T6** bearing six stereogenic centres folds around Eu(III) or Lu(III) to generate an overhand-knot complex **T7** in 85% and 90% yield, respectively. Subsequent RCM with the Hoveyda–Grubbs second-generation catalyst closed the strand ends to give pseudo-D_3_-symmetric lanthanide trefoil knot **T8** in 88% yield for Lu(III) and 90% yield for Eu(III). X-ray diffraction of the Eu(III) complex confirmed the Lambda trefoil topology.

**Figure 6:**
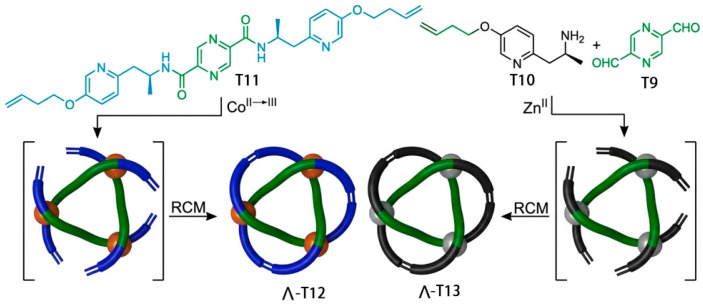
Stereoselective synthesis of enantiopure trefoil knots via trimeric circular helicate assembly and narcissistic self-sorting. Reproduced with permission from Ref. [50], copyright 2019 J. Am. Chem. Soc.

Self-sorting circular helicates have also enabled highly efficient stereoselective trefoil-knot synthesis. Leigh and co-workers reported an imine-Zn(II) system in which a chiral amine **T10**, pyrazine-2,5-dicarbaldehyde **T9** and Zn(BF_4_)_2_ assemble into a homochiral trimeric circular helicate in 90% yield (Figure 6) [50]. RCM then produced the Zn(II)-coordinated trefoil knot **T13** in 98% yield, with up to 90% overall yield over the two assembly steps. A racemic mixture of the chiral amines did not form a statistical distribution but underwent narcissistic self-sorting to give the two enantiomeric helicates. An analogous amide-Co(III) system gave the corresponding circular helicate in 90% yield and the Co(III)-coordinated trefoil knot **T12** in 99% yield. The amplified CD response of the knot, 4.8 times stronger than that of the point-chiral ligand, demonstrates the strong influence of topological chirality on the chromophore environment.

**Figure 7:**
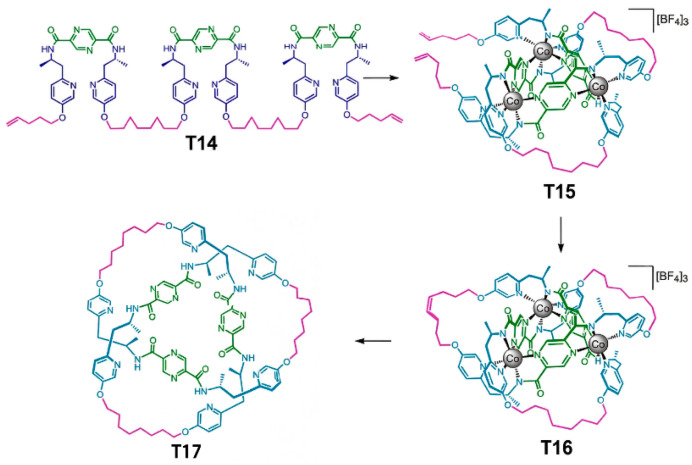
Single-handed trefoil knot formation (**T17**). Reproduced with permission from Ref. [51], copyright 2024 J. Am. Chem. Soc.

Zhong, Leigh, and co-workers translated the circular-helicate concept to the folding of a single molecular strand using Co(II)/Co(III) ions as coordination chaperones (Figure 7) [51]. Labile Co(II) centres first promote error-correcting folding of a tritopic ligand strand into an overhand-knot-like circular helicate; in situ oxidation to kinetically inert Co(III) locks the fold, affording the metalated overhand knot **T15** in 85% yield. RCM then captured the topology to give the Co(III)-coordinated trefoil knot **T16** in 99% yield, and reductive demetallation followed by hydrogenation produced the metal-free trefoil knot **T17** in 35% yield. X-ray crystallography confirmed the stereodefined folded and knotted structures.

**Figure 8:**
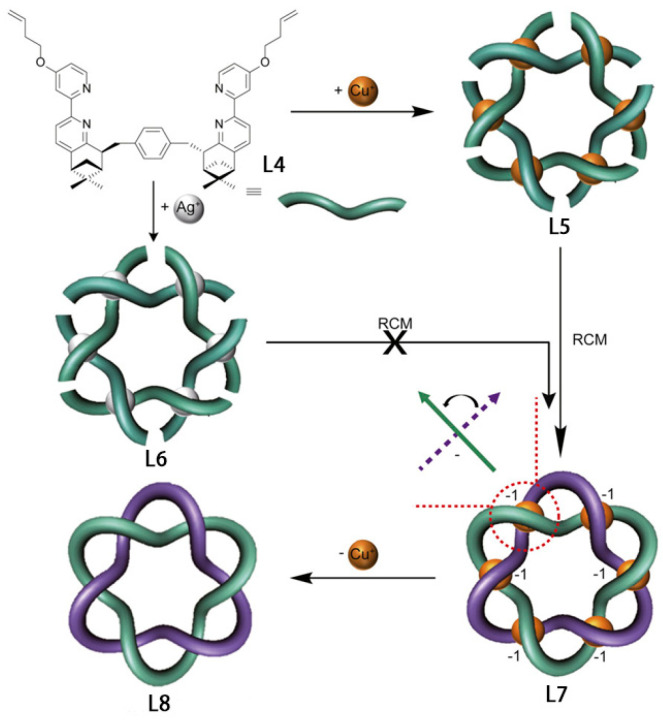
Completely stereoselective synthesis of Star of David [2]catenane. Reproduced with permission from Ref. [52], copyright 2022 Chem.

The same chirality-transfer logic has been extended from knots to topologically chiral links. Zhang and co-workers achieved a stereoselective synthesis of a single-handed Star of David [2]catenane, corresponding to a $6_{1}^{2}$ link (Figure 8) [52]. A CHIRAGEN-type pinene-bipyridine ligand **L4** assembled with Cu(I) into a homochiral hexameric circular helicate **L5** in 98% yield. RCM followed by anion exchange afforded the metallated catenane L7 in 87% yield over two steps, and demetallation with aqueous Na_4_EDTA gave the organic Star of David [2]catenand **L8** in 50% yield. NMR data and ESI-MS fragmentation supported the interlocked topology. Because all six metal centres possess the same configuration, the link contains six crossings of defined handedness and a predetermined writhe of w = −6.

**Figure 9:**
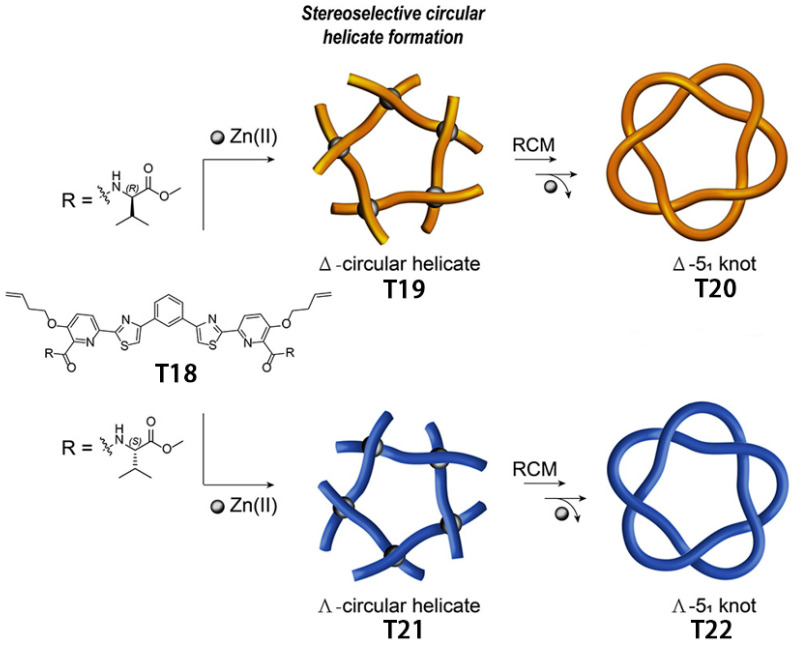
Completely stereoselective synthesis of cinquefoil knot from a pentameric circular helicate. Reproduced with permission from Ref. [53], copyright 2023 Chem.

Zhang and co-workers reported a completely stereospecific synthesis of molecular $5_{1}$ knots through covalent capture of a homochiral pentameric circular helicate (Figure 9) [53]. The D-valine-derived ligand assembled with Zn(OTf)_2_ in acetonitrile to form the Δ helicate **T19** in 95% yield, whereas the opposite ligand enantiomer generated the Λ helicate **T21**. RCM afforded the metallated $5_{1}$ knot in 90% yield, corresponding to an overall two-step yield of approximately 86%, and demetallation with Li_2_S furnished the organic knots in 30–32% yield. The absolute configuration of the valine units predetermines the helicity of the circular helicate, the handedness of the $5_{1}$ knot and the sign of its topological writhe. This work extended stereospecific topological synthesis beyond trefoil knots and demonstrated that point-to-helical-to-topological chirality transfer can be used to access higher-order prime knots.

Collectively, these examples show that circular helicates and folded strands provide a modular route from local stereochemical information to persistent topological handedness. They also demonstrate that increasing topological complexity requires not only stronger templation but also increasingly precise control over pathway, strand sequence, and kinetic fixation.

### 2.2. Template-Free Strategies

Although the vast majority of topological syntheses rely on metal-ion templation, several notable and conceptually innovative examples demonstrate that topologically chiral structures can also be generated under completely template-free conditions. In these systems, folding and entanglement are driven entirely by intrinsic molecular interactions, including hydrophobic effects, π–π stacking, hydrogen bonding [54,55], and dynamic covalent chemistry.

**Figure 10:**
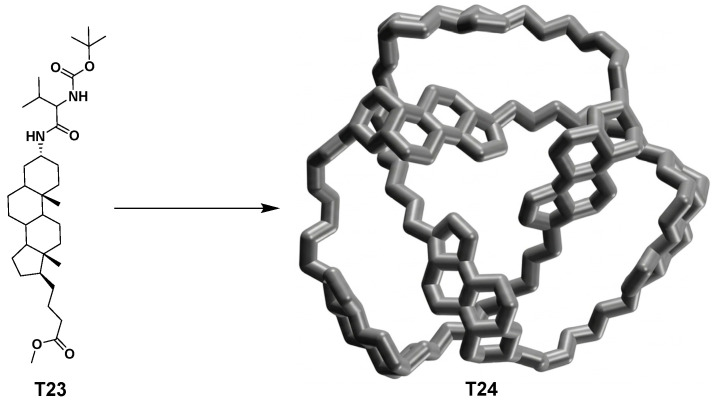
Peptide–steroid trefoil knot formed by template-free macrocyclization. Reproduced with permission from Ref. [56], copyright 2006 Angew. Chem. Int. Ed.

Feigel and co-workers reported one of the earliest non-metal-templated trefoil knots constructed from peptide-like building blocks (Figure 10) [56]. Alternating L-valine and 3α-aminodeoxycholic acid units were assembled into cyclic oligoamides in which the concave steroid framework, intramolecular hydrogen bonding and amino acid chirality promote the required strand crossings. Coupling of protected dipeptide fragments gave the linear hexapeptide precursor in 85% yield; subsequent deprotection, pentafluorophenyl ester activation, and macrocyclization afforded a smaller macrocycle in 32% yield and the trefoil-knot cyclopeptide **T24** in 21% yield. NMR spectroscopy suggested close strand-crossing contacts, and single-crystal X-ray diffraction confirmed the trefoil topology. Only the 3_1_ diastereomer was isolated, indicating that the intrinsic chirality of the amino acid-steroid sequence biases the knot toward a single crossing sense.

**Figure 11:**
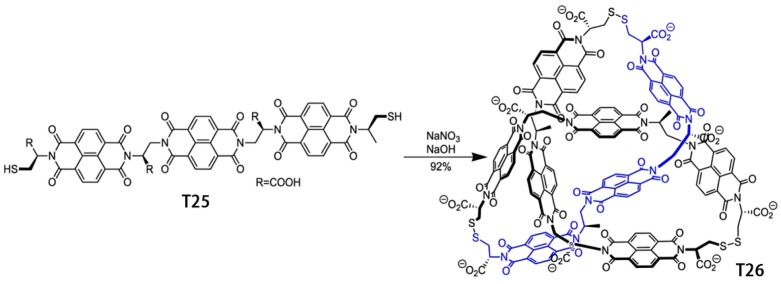
Chemical structure of the right-handed knot. Reproduced with permission from ref.[57], copyright 2012 Science.

A major advance in metal-free stereoselective knot synthesis was reported by Sanders and co-workers, who discovered a purely organic trefoil knot through aqueous dynamic covalent self-assembly (Figure 11) [57]. The amino acid-derived dithiol building block, containing three hydrophobic naphthalenediimide units and hydrophilic amino acid residues **T25**, was prepared in five steps in 62% overall yield. Oxidation in water generated a disulfide dynamic combinatorial library in which increasing the medium polarity with NaNO_3_ amplified the knotted **T26** to 94%; preparative HPLC then afforded the isolated knot in 92% yield. MS excluded a catenane topology, while NMR spectroscopy indicated a compact C_3_-symmetric structure with shielded aromatic resonances. The all-L and all-D building blocks afforded enantiomeric knots, and racemic mixtures largely self-sorted into a racemic pair of homochiral knots.

**Figure 12:**
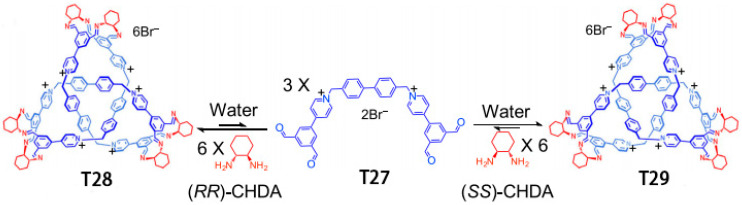
Structural formulae of a pair of enantiomeric trefoil knots. Reproduced with permission from Ref. [58], copyright 2022 Nat. Commun.

Li and co-workers later reported a water-assisted self-templated trefoil knot formed by dynamic imine condensation (Figure 12) [58]. Condensation of a dicationic tetraformyl precursor with chiral 1,2-diaminocyclohexane in water afforded an imine trefoil knot as the only observable product by NMR spectroscopy, although an isolated yield was not reported because isolation or reduction led to degradation. ESI-HRMS confirmed the 3:6 aldehyde/diamine composition, and NMR and CD spectroscopy supported the intertwined topology. The diamine configuration dictates the knot handedness: (SS)-CHDA gives the P knot, whereas (RR)-CHDA gives the mirror-image M knot. In organic solvents, the same components form a topologically trivial macrocycle, demonstrating that water-driven hydrophobic collapse is essential for knot formation.

### 2.3. Coordination-Driven Self-Assembly

Coordination-driven self-assembly plays a central and multifaceted role in the construction of topologically complex architectures, extending far beyond conventional helicate intermediates to include pathway-dependent, stereodirected, and highly entangled assemblies. In these systems, metal ions serve as both structural templates and stereochemical amplifiers and directors [59,60], enabling the formation of sophisticated entanglements that do not follow a single predefined helical pathway.

**Figure 13:**
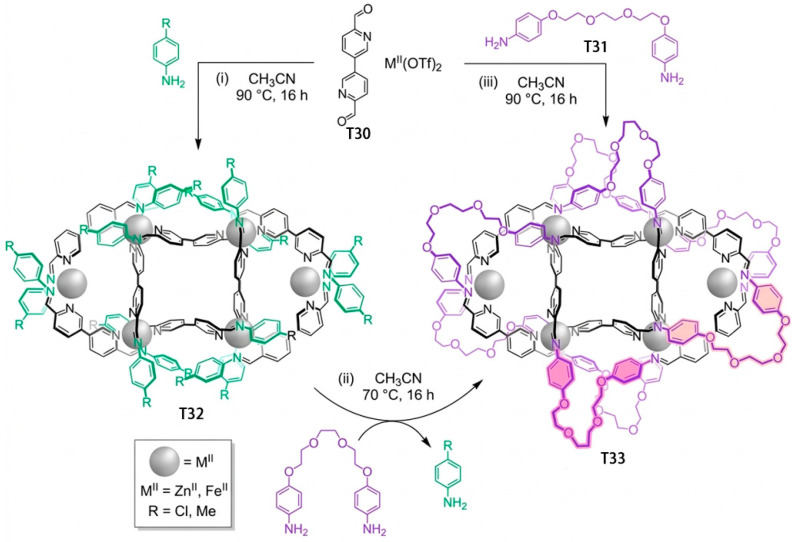
Synthesis of an eight-crossing molecular knot. Reproduced with permission from Ref. [61], copyright 2021 Chem.

Nitschke and co-workers reported stereochemical control over an intrinsically topologically chiral 8_19_ knot using subcomponent self-assembly (Figure 13) [61]. Direct treatment of a dialdehyde **T30**, dianiline and Zn(OTf)_2_ in acetonitrile at 90 °C afforded the metallated knot in 72% yield, while a stepwise aniline-exchange route from a preassembled circular helicate improved the yield to 92%. An analogous Fe(II)-templated knot was also prepared, and reductive demetallation with BH_3_·THF furnished the fully organic 240-atom knotted loop in 62% yield. NMR, HRMS and X-ray crystallography established the 8-crossing topology. Incorporation of enantiopure cyclohexane-based dianilines enabled strong diastereoselective induction, showing that remote covalent stereocentres can bias the metal-centre configurations and the global topological handedness of a high-order knot.

**Figure 14:**
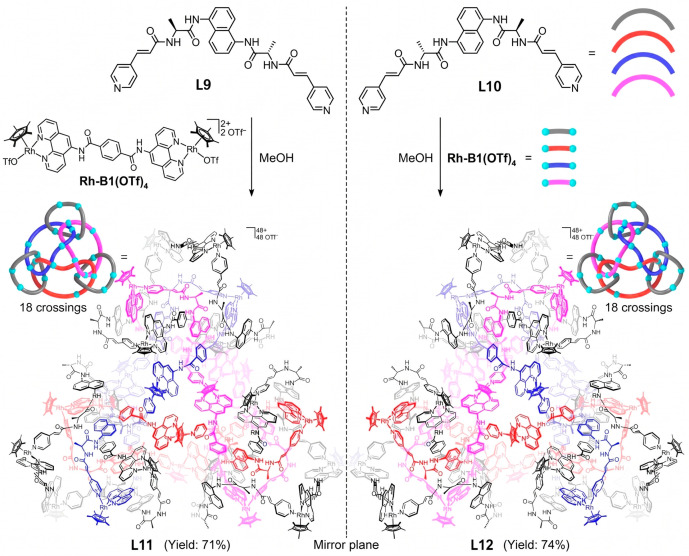
Stereoselective synthesis of topologically chiral $18_{1}^{6}$ links **L11** and **L12**. Reproduced with permission from Ref. [62], copyright 2025 J. Am. Chem. Soc.

The scalability of coordination-driven stereocontrol was further demonstrated by Cui, Jin, and co-workers in the assembly of a topologically chiral [6]catenane containing 18 crossings [62], assigned as an $18_{1}^{6}$ link (Figure 14). Twelve chiral semirigid bidentate ligands bearing alanine residues and twelve binuclear half-sandwich Rh(III) clips assembled in methanol to give **L11** in 71% yield; the opposite ligand enantiomer furnished **L12** in 74% yield. X-ray crystal structure confirmed the six-ring entangled architecture and revealed three cyclic [3]catenane subunits and one closed three-link chain subunit as topologically chiral stereogenic elements. CD spectra confirmed the enantiomeric relationship of the two assemblies.

**Figure 15:**
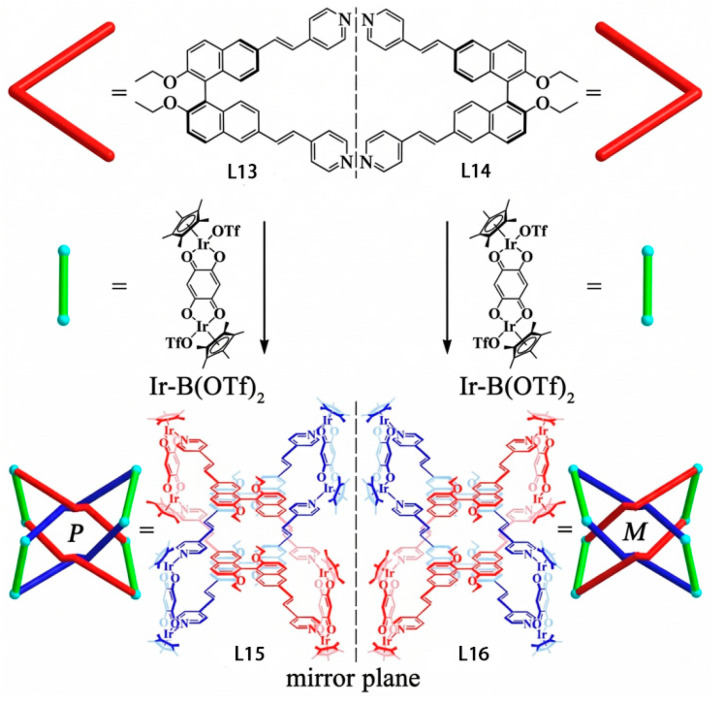
Stereoselective syntheses of topologically chiral Solomon links. Reproduced with permission from Ref. [63], copyright 2020 J. Am. Chem. Soc.

Axial chirality can also serve as a source of stereochemical bias for topological synthesis. Cui, Jin, and co-workers reported the stereoselective synthesis of an enantiopure topologically chiral Solomon link using BINOL-derived axially chiral bispyridyl ligands and a dinuclear iridium (Figure 15) acceptor [63]. X-ray crystal structure confirmed the Solomon link topology, formed by double interlocking of two twisted V-shaped metallacyclic rings through N-Ir coordination bonds. The stereochemical outcome is mainly governed by π–π stacking between the axially chiral ligands. DOSY showed a single discrete assembly, and CD spectra verified the formation of topological enantiomers. This study demonstrates that topological chirality can be induced not only by point chirality but also by axial chirality.

Together, these coordination-driven examples demonstrate that stereoselective topological synthesis is no longer limited to small trefoil knots. By combining dynamic covalent chemistry, metal-centre stereochemistry and component geometry, it is now possible to access high-crossing knots and multi-component catenanes with controlled chirality.

**Figure 16:**
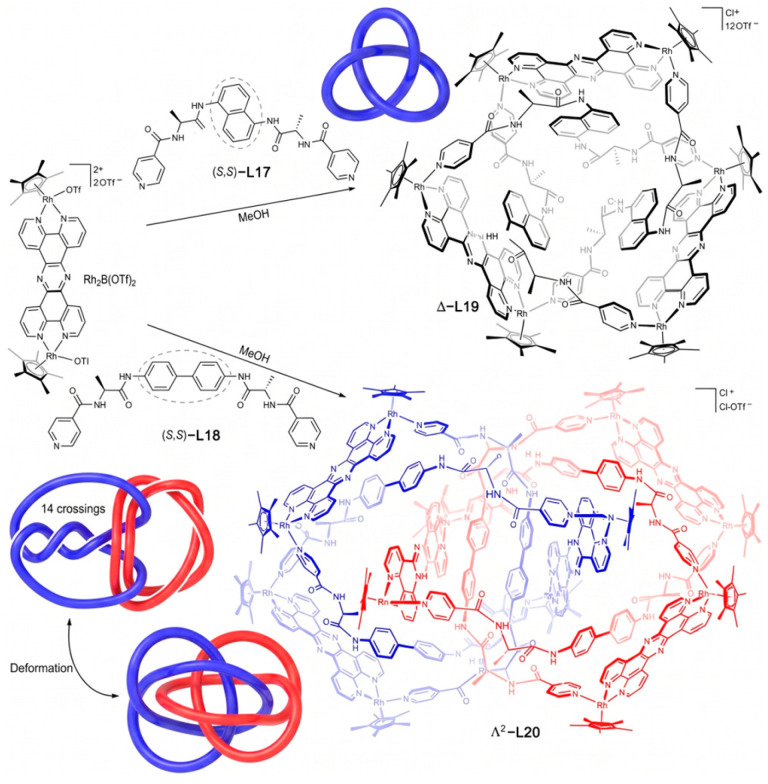
Stereoselective syntheses of topologically chiral trefoil knots and double trefoil links. Reproduced with permission from Ref. [59], copyright 2022 Nat. Synth.

Cui and Jin reported a coordination-driven strategy for constructing a molecular prime link by interlocking two homochiral trefoil knots [59]. Self-assembly of the L-alanine-derived bidentate ligand (S, S)-**L17** with a binuclear half-sandwich Rh(III) clip afforded the right-handed trefoil knot Δ-**L19** in 81% yield, while the corresponding D-alanine ligand gave the mirror-image Λ-**L19** in 86% yield (Figure 16). When the naphthyl spacer was replaced by a longer biphenyl unit, ligand (S, S)-**L18** generated a double trefoil link Λ^2^-**L20** in 78% yield through quadruple interlocking of two left-handed trefoil knots; its enantiomer Δ^2^-**L20** was obtained from (R, R)-**L18** in 75% yield. Single-crystal X-ray diffraction revealed a 14-crossing prime link. The formation of these structures is stabilized by solvophobic effects, π–π stacking and hydrogen-bonding interactions, demonstrating that point chirality in amino-acid-derived ligands can be amplified into high-order topological chirality.

**Figure 17:**
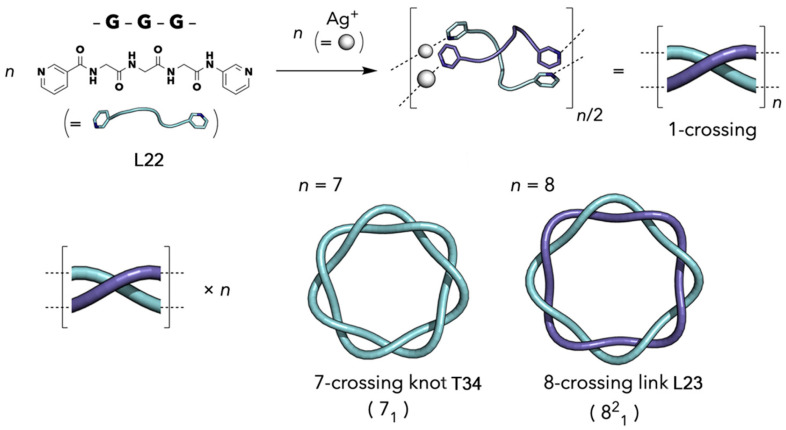
Silver(I)-induced folding and assembly of ditopic triglycine **L22**, which gives 7-crossing torus **T34** and 8-crossing torus **L23**. Reproduced with permission from Ref. [64], copyright 2020 Chem.

Inomata, Sawada, and Fujita demonstrated that flexible short peptides can be converted into highly entangled topological structures through metal-induced folding and assembly [64]. A ditopic triglycine ligand **L22** bearing terminal pyridyl groups assembled with Ag(I) ions in nitromethane to generate a 7_1_ torus knot **T34** and an $8_{1}^{2}$ torus link **L23** (Figure 17) through circular oligomerization of Ag–peptide motifs. The structures were supported by single-crystal X-ray diffraction. Although the GGG ligand gave racemic torus complexes, introduction of an alanine residue into the AGG sequence transferred point chirality into the metal–peptide assembly and afforded enantiomerically pure torus complexes, including an enantiopure $8_{1}^{2}$ link confirmed by X-ray crystallography.

### 2.4. Chirality Self-Sorting

In multicomponent assemblies, stereoselective knot and link formation is often complicated by the presence of multiple homo- and heterochiral pathways. Chiral self-sorting provides a powerful mechanism for improving stereochemical fidelity by directing components into preferred homochiral or heterochiral assemblies [65,66,67].

**Figure 18:**
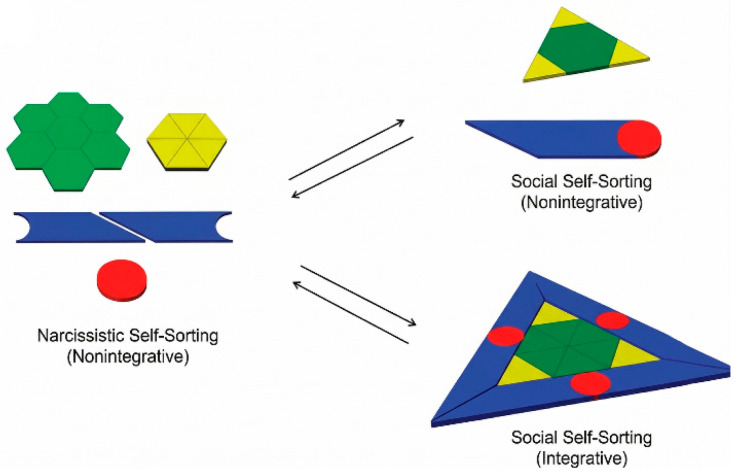
Schematic representation of the different types of self-sorting. Reproduced with permission from Ref. [68], copyright 2026 Trends Chem.

In narcissistic self-sorting, enantiomerically identical components preferentially assemble with one another, producing homochiral architectures rather than statistical mixtures (Figure 18). In topological synthesis, this process can determine whether racemic precursors give separable pairs of enantiomeric knots or complex mixtures of diastereomeric assemblies.

**Figure 19:**
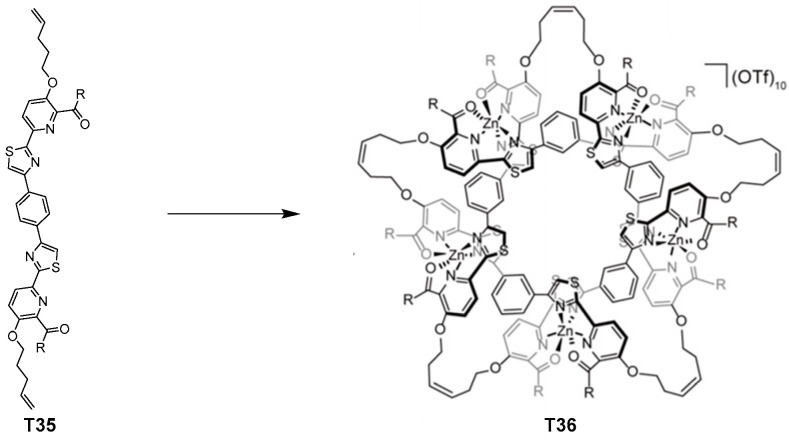
Amino acid–directed chiral self-sorting and stereocontrol in cinquefoil knot assembly. Reproduced with permission from Ref. [44], copyright 2026 Chem.

Chiral self-sorting provides a powerful strategy for regulating stereochemical outcomes in topologically complex systems, particularly when multiple stereoisomeric pathways are accessible. A recent study by Zhang and co-workers [44] demonstrated that exquisite control over chiral self-sorting can be achieved through biomimetic site-specific modification of ligand strands in a synthetic cinquefoil knot system (Figure 19). Incorporation of amino acid and dipeptide motifs into ligand frameworks introduces tunable noncovalent interactions including π–π stacking, CH–π interactions, and hydrogen bonding. While single amino acid residues typically induce only weak stereochemical bias, dipeptide-functionalized systems exhibit strong, high-fidelity narcissistic self-sorting, shifting the assembly from kinetic to thermodynamic control and yielding highly stereochemically pure topological products **T36** in 90% yield.

**Figure 20:**
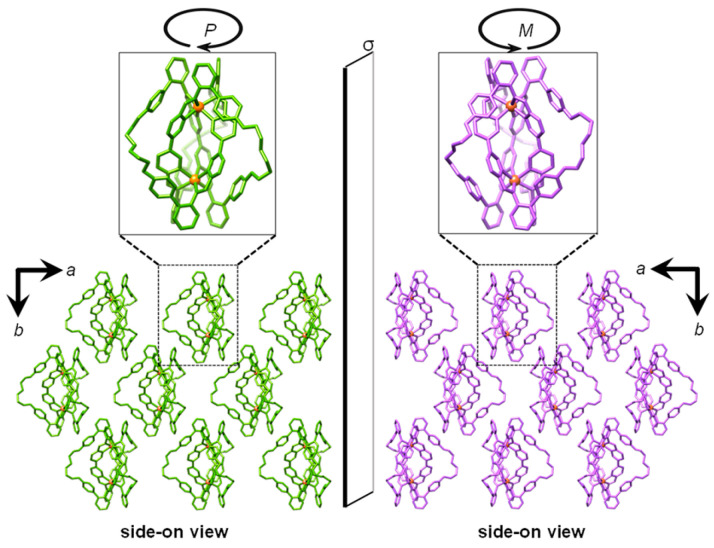
Contra-helical trefoil knots and crystallization-driven chiral self-sorting. Reproduced with permission from Ref. [69], copyright 2023 Nat. Synth.

Stereochemical amplification can also occur during crystallization. Wu, Liu, and co-workers introduced a nested contra-helical strategy for Fe(II)-templated trefoil knots (Figure 20) [69]. Condensation of bipyridyl dialdehydes and diamines in the presence of Fe(OTf)_2_, followed by anion exchange, afforded a series of torus-knot complexes in 90–93% yield. The linker length controls topomechanical strain and thereby modulates the spin-crossover behaviour of the Fe(II) centres: more strained knots undergo thermally induced spin crossover, whereas a less strained analogue remains mainly high spin. Racemic TK undergoes narcissistic chiral self-sorting during crystallization, allowing manual separation of single crystals of the two topological enantiomers and assignment of their CD spectra and optical rotations.

## 3. Applications of Stereodefined Topological Structures

The development of stereochemically defined molecular knots, links, and catenanes has begun to reveal how molecular topology can influence functional behaviour. Topological chirality can provide configurationally stable chiral frameworks, while mechanically interlocked architectures introduce co-conformational dynamics, mechanically constrained environments, and shape-persistent cavities. These features make stereodefined topological structures attractive platforms for exploring structure–function relationships beyond conventional covalent molecules.

Although the stereoselective synthesis of topologically chiral molecular knots and links has advanced rapidly, their practical applications remain relatively underexplored compared with their synthetic development. Reported examples mainly involve host–guest binding, chiral recognition, catalysis, and spin-selective charge transport. In this section, representative applications are discussed with emphasis on how topological chirality, molecular entanglement, and confined chiral environments contribute to functional performance.

### 3.1. Host–Guest Function

Topological entanglement provides a powerful mechanism for regulating host–guest behaviour by introducing mechanical gating and steric hindrance to guest ingress and egress. Covalently linked knotted cage frameworks provide a striking demonstration: interwoven topology mechanically “locks” guests inside the internal cavity and drastically slows guest exchange kinetics, opening new opportunities in molecular confinement, guest retention, and stimuli-responsive controlled release.

**Figure 21:**
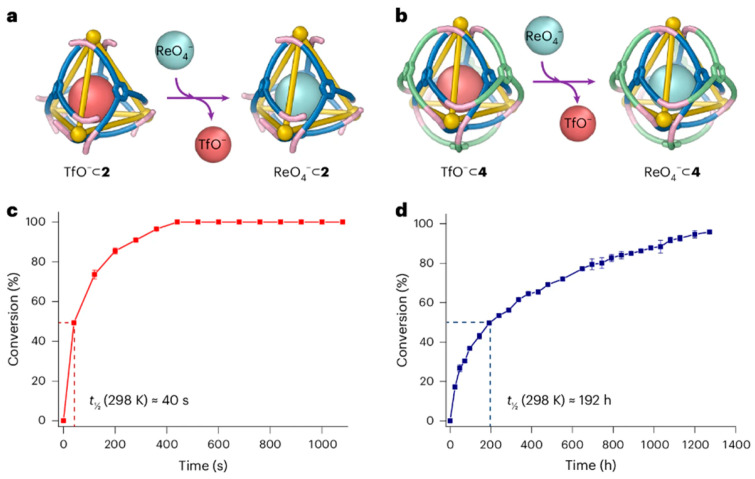
Covalently linked knotted cages and mechanically gated guest exchange. Schematic illustrations of the displacement of TfO^−^ by ReO_4_^−^ within **2** (**a**) and **4** (**b**). (**c**,**d**) show the traces of these processes within **2** and **4**. Reproduced with permission from Ref. [70], copyright 2025 Nat. Synth.

A striking demonstration was provided by Nitschke and co-workers [70], who reported a streamlined one-pot subcomponent self-assembly strategy that converts predesigned cage frameworks into covalently linked knotted cage frameworks (Figure 21). In this system, a topologically chiral trefoil tetrahedron mechanically traps guests inside its central cavity, resulting in an extraordinary kinetic effect: guest exchange is slowed by a factor of approximately 17,000 relative to the analogous non-interwoven tetrahedral cage. Furthermore, these assemblies can be reduced and demetallated to yield metal-free organic interwoven structures while retaining topology-enhanced robustness and guest-binding properties. This work provides a clear blueprint for using architectural entanglement as a purely mechanical design element to modulate molecular confinement, guest retention, and controlled-release behaviour.

Complementarily, homochiral metal–organic cages assembled from stereochemically inert, chiral metalloligands have been successfully applied to the enantioseparation of atropisomeric guests in aqueous media, illustrating how stereodefined chiral cavities enable practical enantioselective recognition, discrimination, and recyclable resolution.

### 3.2. Catalysis

**Figure 22:**
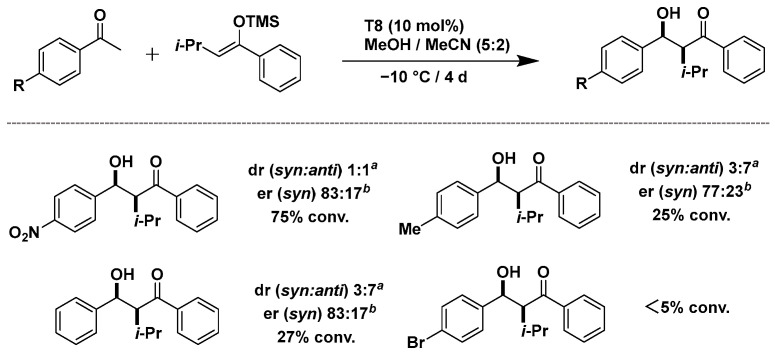
Europium-ligand-catalyzed Mukaiyama aldol reactions. Chiral trefoil knot **T8** catalyzed asymmetric Mukaiyama aldol reactions. ^a^ Determined by ^1^H NMR analysis. ^b^ Determined by chiral HPLC. Adapted with permission from Ref. [16], copyright 2016 J. Am. Chem. Soc.

A particularly important demonstration of the functional relevance of topological chirality was provided by Leigh and co-workers [16], who employed a single-handed trefoil knot as a chiral catalyst for asymmetric transformations. The knot, obtained via lanthanide-templated folding and covalent capture, coordinates a lanthanide(III) ion within a topologically defined chiral pocket, which serves as a Lewis acidic catalytic centre. In Mukaiyama aldol reactions, the corresponding Eu(III) knot complex delivered enantioselectivities of up to 83:17 er (Figure 22, significantly outperforming analogous unknotted ligand systems. Mechanistic studies indicate that, although the metal centre remains accessible to substrates, the continuous covalent backbone of the knot enforces a well-defined chiral environment, thereby enhancing stereocontrol during the reaction. This work represents the example of a molecular knot being utilized in asymmetric catalysis, and clearly illustrates how topological entanglement can be translated into functional stereochemical control.

In this catalytic system, the lanthanide-containing trefoil knot functions as a cationic Lewis acid catalyst. The positive charge associated with the metal complex facilitates substrate activation, while the continuous knotted backbone creates a confined chiral environment around the accessible metal centre. The Mukaiyama aldol reaction proceeded with measurable conversion under the reported conditions, and the knotted catalyst delivered higher enantioselectivity than structurally related unknotted controls. This comparison indicates that the catalytic performance is governed not only by the Lewis acidity and charge state of the metal centre, but also by the stereochemically defined topology of the knotted ligand framework. Beyond asymmetric Mukaiyama aldol catalysis, molecular knots have also been used to regulate catalytic activity through topological confinement and allosteric control. Leigh and co-workers demonstrated that a molecular pentafoil knot can allosterically initiate and regulate catalysis, showing that knot topology can influence substrate access and catalytic switching [34]. Trabolsi and co-workers further showed that metal–organic trefoil knots can promote C–Br bond activation [17]. These examples indicate that molecular knots can function not only as chiral catalysts but also as topology-defined catalytic microenvironments.

### 3.3. Chiral Recognition and Enantioseparation

**Figure 23:**
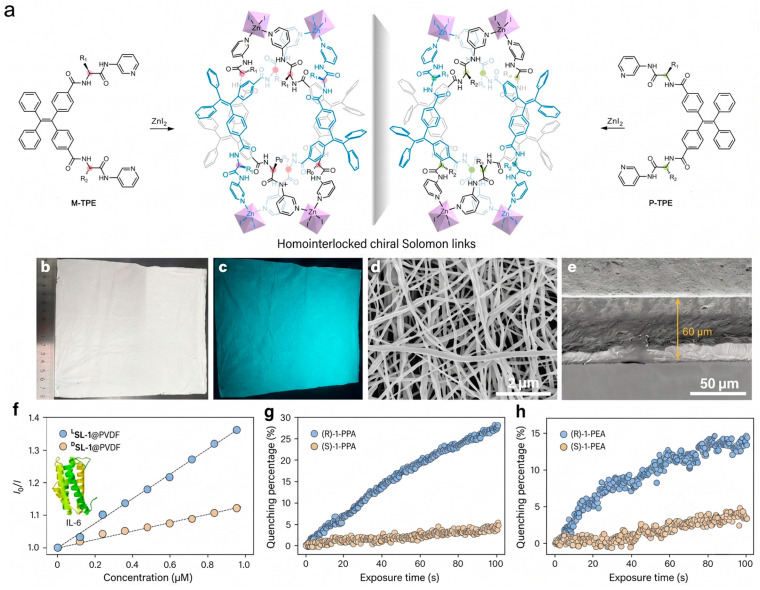
Amino-acid-encoded chiral Solomon links for enantioselective recognition and sensing. (**a**) Design and self-assembly of amino-acid-encoded chiral Solomon links. (**b**–**e**) Photographs, fluorescence images, and SEM images of Solomon-link-containing PVDF composite membranes. (**f**) Stern–Volmer plot for fluorescence sensing. (**g**,**h**) Time-dependent fluorescence quenching responses toward chiral amines. Reproduced with permission from Ref. [71], copyright 2026 Nat. Synth.

Yang, Cui, Davis, and co-workers developed an amino-acid-encoded approach to programmable chiral Solomon links with multilevel chirality and functional recognition (Figure 23) [71]. Tetraphenylethylene-based pseudopeptide ligands bearing L- or D-amino acid residues assemble with ZnI_2_ to form doubly interlocked [2]catenanes (Figure 23a) in 60–65% isolated yield. X-ray crystallography, NMR, and MALDI-TOF-MS confirmed four-crossing Solomon link topologies stabilized by hydrogen bonds, π–π stacking and C–H···π interactions. The amino acid residues define ligand chirality, TPE helicity, metallacyclic-ring handedness and topological chirality, leading to strong chiral amplification: molar optical rotations are 79–348 times larger than those of the parent ligands. The tunable cavities bind peptides with association constants up to 5.6 × 10^4^ M^−1^ and enantioselectivity factors up to 12.7. Incorporation into PVDF membranes enabled fluorescence sensing (Figure 23b–e) of interleukin-6 with a detection limit of approximately 11.5 nM (Figure 23f) and enantioselective detection of chiral amines (Figure 23g,h).

**Figure 24:**
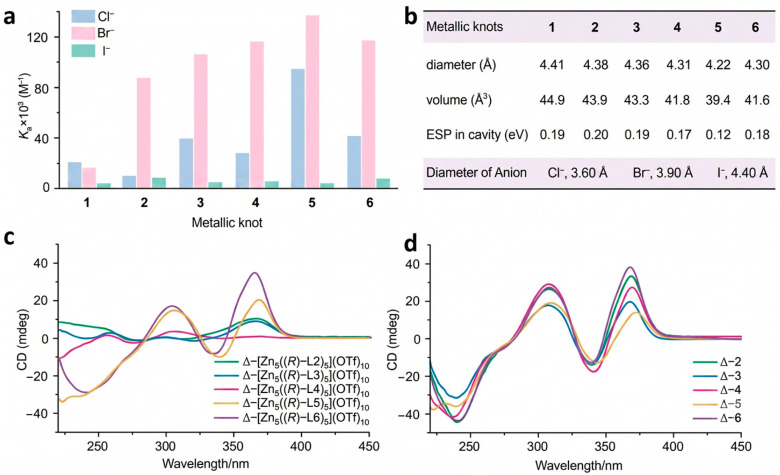
Site-specific amino-acid modification regulates the recognition and chiroptical properties of cinquefoil knots. (**a**) Binding constants of amino-acid-modified cinquefoil knots toward selected halides obtained from UV–vis titration in acetonitrile. (**b**) Calculated cavity diameter, volume, and electrostatic potential of the corresponding metallic knots. (**c**) CD spectra of the open circular helicates. (**d**) CD spectra of the closed 5_1_ knots. Reproduced with permission from Ref. [44], copyright 2026 Chem.

Yang, Zhang, and co-workers further demonstrated that biomimetic site-specific modification can be used not only to regulate chiral self-sorting, but also to tune the recognition and chiroptical properties of synthetic 5_1_ knots [44]. Importantly, these peripheral amino-acid motifs remotely modulated the central cavity of the knots. UV–vis titrations showed selective halide binding in the order Br^−^ > Cl^−^ > I^−^, with the Phe–Phe-modified knot displaying the strongest Br^-^ affinity (K_a_ = 1.37 × 10^5^ M^−1^) (Figure 24a), approximately eight times higher than that of the parent valine knot. Computational analysis indicated that the amino-acid substituents alter the cavity size, volume, and electrostatic potential, suggesting that peripheral modification can fine-tune recognition inside a topologically constrained cavity (Figure 24b). CD spectroscopy further showed that the closed 5_1_ knots display similar and generally stronger chiral responses than their open helicates, indicating that the knotted topology dominates the chiral environment experienced by the chromophores (Figure 24c,d). This work therefore establishes site-specific biomimetic modification as a practical strategy for engineering recognition and chiroptical functions in topologically complex molecular knots.

### 3.4. Spin-Selective Transport and Spintronics

**Figure 25:**
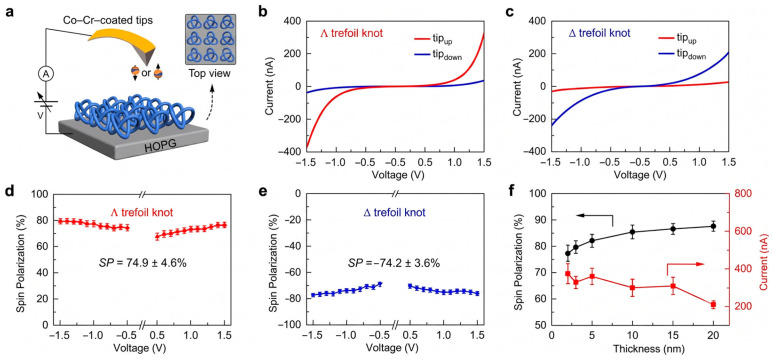
AFM measurements. (**a**) Schematic illustration of an AFM setup. (**b**,**c**) Current−voltage curves of the Λ (**b**) and Δ (**c**) molecular trefoil knot thin films measured by AFM at room temperature. The current−voltage curves were measured 50 times at different spots on the substrate. The lines represent the average results. (**d**,**e**) Spin polarization as a function of the applied bias for Λ (**d**) and Δ (**e**) molecular trefoil knots. Spin polarization was calculated based on the results shown in (**b**,**c**). (**f**) Thickness-dependent absolute spin polarization and absolute current intensities at ±1.5 V. Reproduced with permission from Ref. [20], copyright 2023 J. Am. Chem. Soc.

Yang and co-workers demonstrated that topologically chiral molecular trefoil knots can serve as efficient spin filters for CISS applications [20]. Unlike conventional chiral molecules containing stereogenic carbon centres or helically chiral backbones, the amide-based trefoil knots used in this study possess no classical stereogenic units; their chirality arises solely from the spatial arrangement of the over-and-under crossings in the knot. The racemic knots were separated by chiral HPLC to afford the Λ and Δ enantiomers, whose opposite topological handedness was confirmed by mirror-image CD spectra and X-ray structures. Thin films of the enantiopure knots retained their chiroptical activity, as shown by solid-state CD and PM-IRRAS measurements, indicating that the topological chirality is preserved after film formation. Magnetic conductive-probe AFM measurements revealed strong handedness-dependent charge transport: the Λ and Δ knot films showed opposite spin-polarization signs (Figure 25a–c), with spin polarizations of 74.9 ± 4.6% and −74.2 ± 3.6% (Figure 25d,e), respectively, and values reaching up to 88% with increasing film thickness (Figure 25f). The currents were about two orders of magnitude higher than those typically observed for DNA, oligopeptides, or helicenes under comparable bias, giving an exceptionally high figure of merit for a small-molecule chiral spin filter. Spin-valve measurements further confirmed opposite magnetoresistance responses for the two knot enantiomers, and the spin-selective behaviour remained stable even after heating at 350 °C for 2 h in air. These results suggest that topological chirality offers a robust and orientation-insensitive platform for CISS, while the compact, conjugated, and mechanically constrained knot framework may facilitate efficient spin-selective transport through electron–electron interactions. This work therefore expands the functional scope of molecular knots from recognition and catalysis to spintronic materials.

Mechanistic insight into this behaviour has been further advanced by a theoretical framework for trefoil-knot CISS that emphasizes two central ingredients—asymmetric multiple transport channels and geometric spin–orbit coupling—and explicitly connects the persistence of high spin polarization to preservation of knot topology [20]. In this model, progressive “topology suppression” that removes nontrivial knot character leads to a marked drop in spin polarization, supporting the conclusion that ultrahigh spin selectivity is strongly correlated with the underlying knot topology. The analysis also suggests that knot-based CISS can remain stable under substantial structural distortions, providing a rationale for experimentally observed high-temperature stability. Collectively, these studies indicate that stereodefined topological chirality is not merely compatible with spin-selective transport, but may provide a route to robust spin filtering elements where performance is less sensitive to geometric perturbations than in conventional structurally chiral systems.

## 4. Conclusions

Topological chirality represents a fundamentally unique form of molecular asymmetry, encoded entirely within the global connectivity and mechanical entanglement of molecular frameworks. Recent advances have transformed the field from isolated structural demonstrations of knotted molecules to stereoselective, stereospecific, and even programmable synthetic systems capable of reliably producing enantiopure knots and interlocked links. Importantly, stereodefined topological architectures are now rapidly evolving into functional materials with demonstrated utility in chiroptical devices, spin-selective transport, host–guest chemistry, asymmetric catalysis, and enantioselective sensing.

Despite extraordinary progress, several key challenges remain: (1) limited generality and substrate scope of existing synthetic platforms; (2) scarcity of broadly applicable, scalable, and environmentally benign metal-free strategies; (3) a critical need for systematic, quantitative topology–property–function correlations; and (4) difficulty in constructing increasingly complex topologies with complete stereocontrol. Overcoming these challenges will unlock the full scientific and technological potential of topological chirality, paving the way for a new generation of mechanically robust, stereochemically persistent, and functionally superior supramolecular materials and devices.

## Data Availability

No new data were generated or analysed in this review. Data sharing is not applicable to this article.
